# Supplementary material for: Brain glucose and ketone metabolism in first-episode psychosis: Neuroimaging and brain metabolism before and after antipsychotic treatment: The protocol for the CAST-ATP study
Source: PLoS One. 2025 Jun 30;20(6):e0325489. doi: 10.1371/journal.pone.0325489 (PMC12208466; doi:10.1371/journal.pone.0325489)
Supplement: S1 File — (PDF) [file pone.0325489.s001.pdf]

Impacts of Psychosis and Antipsychotics on Cerebral Energy Metabolism: The ATP Project

Impacts de la psychose et des antipsychotiques sur le métabolisme énergétique cérébral : le projet ATP (Antipsychotique-TEP-Psychose)

Original version : V1.3 : 2024-09-27 – English translation: 2024-09-27

Protocol REB CIUSSS de l'Estrie-CHUS: 2025-5589

Final approbation 2024-10- 04

Table of content

1 Information on the team and the clinical study ... 3
2 Abbreviations... 4
3 Project Rationale and Literature Review ... 5
4 RESEARCH QUESTIONS AND OBJECTIVES ... 7
5 Study Population ... 8
5.1 Sample Size ... 8
5.2 Inclusion Criteria ... 8
5.3 Exclusion Criteria ... 9
5.4 Withdrawal Criteria During the Study ... 9
5.5 Concomitant Medication ... 9
6 Research Methodology ... 10
6.1 Study Duration ... 10
6.2 Recruitment ... 10
6.3 Summary of the study ... 10
6.4 Study Procedures ... 11
6.5 Additional Details ... 12
6.6 Detailed Procedures ... 12
6.7 Participant Collaboration ... 14
6.8 Radiotracers Used ... 14
6.9 Participant Withdrawal ... 14
7 Ethical Considerations ... 14
7.1 Risks ... 14
7.2 Benefits ... 15
7.3 Compensation ... 16
7.4 Information and Consent Form ... 16
7.5 Voluntary Participation ... 16
7.6 Significant Incidental Findings ... 16
7.7 Confidentiality ... 16
7.8 Storage of Samples and Data ... 17
7.9 Insurance ... 17
7.10 Clinical Trial Registration ... 17
7.11 Study Limitations ... 17
8 Variables and analysis ... 18
8.1 Analyses ... 18
8.2 List of variables ... 19
9 Data Quality, Safety, and Monitoring ... 21
9.1 Good Clinical Practices ... 21
9.2 Data Management and Validation ... 21
9.3 Quality, Safety, and Ethical Management ... 21
10 Adverse Events Management ... 21
11 Potential Impact and Benefits of the Project ... 22
12 References ... 23

1 Information on the team and the clinical study

Co-Investigators:
- Stephen Cunnane, PhD
  Department of Endocrinology, Department of Medicine
  Faculty of Medicine and Health Sciences
  University of Sherbrooke
  Research Center on Aging
  Integrated University Health and Social Services Center of Estrie – University Hospital of Sherbrooke (CIUSSS de l'Estrie-CHUS)

- Kevin Zemmour, MD
  Medical Psychiatrist, Medical Director of the First Episode of Psychosis Clinic
  Assistant Professor, Department of Psychiatry, Faculty of Medicine and Health Sciences
  University of Sherbrooke

- Maggie Hahn, MD
  Psychiatrist, Psychiatry University of Toronto
  Center for Addiction & Mental Health

Clinical Trial Location (Monocentric):
- CIUSSS de l'Estrie-CHUS
  University Hospital Hôtel-Dieu de Sherbrooke (CHUS HD)

- Research Center on Aging (CDRV)
  Hospital and Accommodation Center D'Youville

- Research Center of the University Hospital of Sherbrooke (CRCHUS)

Collaborating Physicians in the Study:
- Éric Turcotte, MD- University of Sherbrooke - FMSS, Department of Nuclear Medicine and Radiobiology
- Sylvain Grignon, MD, PhD- University of Sherbrooke - FMSS, Department of Psychiatry
- Jean-Daniel Carrier, MD, PhD- University of Sherbrooke - FMSS, Department of Psychiatry

CAST Collaborators (project connecting Copenhagen, Aarhus, Sherbrooke, Toronto):
- Bjorn H. Ebdrup
  Clinical Professor, Department of Clinical Medicine
  University of Copenhagen, Denmark

- Esben Sondergaard
  Clinical Associate Professor, Department of Clinical Medicine
  SDCA-Steno Diabetes Center Aarhus, Aarhus University, Denmark

Other Collaborators:
- Kevin Whittingstall- Professor, FMSS, Department of Diagnostic Radiology - University of Sherbrooke
- Stéphanie Dubreuil- Research Center of CHUS, CIUSSS de l'Estrie-CHUS

Funding:
- Baszucki Brain Research Fund and Ketotherapeutics Research Chair of the University of Sherbrooke.

2 Abbreviations

AP: Antipsychotic
AUDIT: Alcohol Use Disorders Identification Test
BACS: Brief Assessment of Cognition
BAS: Barnes Akathisia Scale
BPRS: Brief Psychiatric Rating Scale
CDSS: Calgary Depression Scale for Schizophrenia
CGI-S: Clinical Global Impression Severity
CIMS: Centre for Molecular Imaging of Sherbrooke
DSM V-TR: Diagnostic and Statistical Manual of Mental Disorders, 5th Edition, Text Revision
DUDIT: Drug Use Disorders Identification Test
DUP: Duration of Untreated Psychosis
PEP Team: First Episode of Psychosis Team
ESS: Epworth Sleepiness Scale
FCQ: Food Craving Questionnaire
FTND: Fagerström Test for Nicotine Dependence
GAF: Global Assessment of Functioning
MARS: Medication Adherence Rating Scale
ATP Project: Pilot Research Project Antipsychotic-PET-Psychosis
SIMPAQ: Simple Physical Activity Questionnaire
UKU: Side Effect Rating Scale

3 Project Rationale and Literature Review

The ATP Research Project (Antipsychotic-PET-Psychosis)

We propose to evaluate the early impacts of psychosis and antipsychotic medications (APs) on brain metabolism (glucose and ketones) in young adults recently diagnosed with a first episode of psychosis.

To do this, we will measure brain and systemic glucose and ketone metabolism before and 4 to 6 weeks after the prescription of APs.

Psychosis

Psychosis, particularly schizophrenia, is one of the most severe and difficult-to-treat mental disorders. It constitutes a global public health issue, as just under 1% of people suffer from psychosis, yet they experience severe consequences that result in a significant economic burden on society.

The diagnosis of psychosis is based on a combination of clinical arguments without relying on biomarkers, especially without signatures in brain imaging. To maintain a certain sensitivity and specificity in diagnosis, Canadian clinicians use a criterion-based manual, DSM-5-TR (American Psychiatric Association 2022). Symptoms are divided into three subcategories: positive symptoms (hallucinations, delusions, disorganization of thought and behavior), negative symptoms (alogia, affective blunting, avolition, anhedonia, asociality), and cognitive symptoms (alterations in concentration, attention, executive functions, working memory, and learning). According to expert consensus and the biopsychosocial reference model, psychosis results from a combination of biological, environmental, and psychological vulnerability factors.

The reference treatment for psychosis also follows the biopsychosocial model, with the success criterion being the improvement of symptomatology and functionality. In the biological aspect of this model, pharmacotherapy, mainly second- and third-generation APs, is the reference treatment. Alongside the biological aspect, psychosocial interventions, notably supporting the person in functional rehabilitation, are added.

Concerning the early stage of psychosis, patients experiencing a first episode of psychosis face a poor prognosis if not quickly and effectively managed, and if relapses are not prevented: learning to live with chronic psychotic symptoms, reduced treatment response, brain function decline, and ultimately, a decrease in quality of life and a loss of more than 10 to 20 years of life expectancy.

Psychotic Disorders and Metabolic Impacts

People with psychotic disorders are twice as likely to die from cardiovascular accidents and have a fourfold higher risk of obesity. APs have significant metabolic side effects, such as weight gain and altered glucose and lipid homeostasis. This increases the risk of developing obesity, type 2 diabetes, and cardiovascular diseases. Moreover, some researchers have shown a positive association between symptom improvement after taking APs and metabolic disturbances, although this link remains controversial. Nonetheless, this suggests common mechanisms between the action of APs and their side effects.

It is generally accepted that interventions aimed at combating these metabolic side effects (notably by prescribing oral antidiabetics to improve insulin sensitivity) do not worsen psychiatric symptoms.

Regarding nutritional interventions, although this is a promising field, few interventions focus on the evaluation and management of the nutritional and metabolic aspects of these patients. Furthermore, weight gain induced by APs is one of the most burdensome side effects and often leads to discontinuation of treatment.

It is important to note that patients experiencing a first episode of psychosis already exhibit certain glucose homeostasis disturbances, such as insulin resistance or prediabetes, even before starting APs. This metabolic disruption could, therefore, be intrinsic to psychotic disorders, independent of the metabolic effect of APs. A meta-analysis comparing AP-naïve patients with glucose dysregulation without psychiatric illness concluded that both groups exhibit a similar gene expression signature. A second meta-analysis focusing on obesity markers also demonstrated a difference between healthy controls and AP-naïve psychotic patients experiencing a first episode of psychosis. Several aspects of brain energy metabolism may be disrupted in psychotic disorders, such as insulin and glucose signaling, glycolysis, astrocyte-neuron coupling, and ATP production by brain cells.

Studies have also linked negative and cognitive symptoms to glucose metabolism dysfunction in the prefrontal cortex, particularly in patients with schizophrenia and who are AP-naïve.

However, according to our review of the literature, there is no data regarding ketone metabolism, the brain's main alternative fuel to glucose, in patients with psychosis.

Some authors have thus proposed that psychotic disorders could be seen as a disruption of systemic and brain energy metabolism. Moreover, these metabolic disruptions could be intrinsic to psychotic disorders, just like the psychiatric symptoms that define these diseases. Thus, the pathophysiology of psychotic disorders could contribute to glucose dysregulation in addition to the deleterious effect of APs. Hence the importance of studies measuring brain metabolism (glucose and ketones) before and after the prescription of APs. However, more data is needed to fully understand the mechanisms related to these disruptions, particularly at the onset of the disease and especially in the brain itself.

It is also important to note that both psychosis and metabolic disorders, such as obesity, dyslipidemia, and type 2 diabetes, are associated with cognitive disorders. Their exacerbation during aging has been little studied and remains to be clarified.

The Program for First Episodes of Psychosis

The CIUSSS de l'Estrie-CHUS and its psychiatry department have their own PEP (First Episode Psychosis) program, known as the PEP Team of Estrie. One of the unique features of accessing this team is that it does not require a medical consultation request. This allows for accelerated follow-ups, with a team leader who analyzes all requests within 24 to 72 hours, performs an initial screening of the requests, and then refers them to a psychiatrist. The timeframe for conducting a psychiatric evaluation is approximately 7 to 10 days. If the person meets the program's eligibility criteria, a joint follow-up between the psychiatrist and a psychosocial worker will begin.

PEP programs offer flexible multidisciplinary follow-up for up to 3 years. To benefit from a PEP team, participants must have never been treated for psychosis for more than 12 months. This specialized program aims to reduce the duration of untreated psychosis and minimize its effects, enabling a return to normal functional development and optimal, sustained recovery over time.

Medical Imaging for the Study of Metabolism

Magnetic resonance imaging (MRI) is the gold standard for imaging psychiatric disorders, but it is mainly used to exclude neurological disorders. In clinical practice, brain imaging is not routinely recommended for people with psychosis that appears before the age of 45, unless there are clinical signs suggesting a neurological problem. As explained earlier, there is no imaging signature or biomarker for psychosis.

To study brain energy metabolism, the addition of quantitative positron emission tomography (PET) with metabolic radiotracers for glucose and ketones (FDG-glucose, 11C-Acetoacetate, 11C-Acetate, etc.) allows for much greater insights. Our team in Sherbrooke was the first to develop the 11C-Acetoacetate tracer to visualize brain ketone uptake, and has thus developed expertise in multi-imaging approaches, combining multi-tracer PET with various MRI modalities (volumetric, functional, diffusion, etc.). Our 15 years of experience in metabolic medical imaging has allowed us to establish a comprehensive multimodal database, including controls of young adults in the same age group as individuals experiencing a first episode of psychosis.

These tools have enabled us to demonstrate in older individuals that, unlike the energy deficit in glucose, ketone uptake and utilization do not decrease with age or with the onset of cognitive decline. Furthermore, increasing plasma ketones through ketogenic supplementation can boost brain fuel supply and largely compensate for the energy deficit in individuals with mild cognitive impairment, the prodromal stage of Alzheimer's disease. This fuel supply could even have a functional impact by improving various cognitive functions. Other conditions with metabolic dysregulation, such as Parkinson's disease and other medical conditions (heart failure, polycystic ovary syndrome, renal failure, etc.), have been or are being studied by our team to better understand the energy deficit in different organs in various diseases, inspiring the development of innovative therapeutic approaches.

To our knowledge, no PET study of brain energy metabolism (glucose and ketones) has been conducted in patients experiencing a first episode of psychosis.

Conclusion

Our expertise in medical imaging will allow us to evaluate and quantify changes in brain energy metabolism at the onset of psychotic illness, before and after the introduction of APs, thereby better understanding the differentiated impacts of the disease and medication on metabolic disturbances. This improved understanding may eventually lead to the development of better treatments for these patients, encompassing the metabolic aspects of the disease.

4 Research questions and objectives

Research Question and Hypothesis

To evaluate the effect of psychosis and the initiation of antipsychotic medication during a first episode of psychosis on brain energy metabolism. The hypothesis is that patients with a first episode of psychosis (PEP) will exhibit frontal glucose hypometabolism, and we expect this to worsen with antipsychotic medication. We expect no changes in ketone metabolism.

Primary Objective

In patients with a first episode of psychosis, evaluate the effect of 4 to 6 weeks of antipsychotic medication on brain metabolism measured by PET scan (cerebral uptake of 11C-Acetoacetate + 18 Fluorodeoxyglucose).

Secondary Objectives

1) In patients with a first episode of psychosis, compare the effect of 4 to 6 weeks of antipsychotic medication on brain metabolism measured by PET scan (cerebral uptake of 11C-Acetoacetate + 18 Fluorodeoxyglucose) to clinical improvement (measured by the percentage change in the Brief Psychiatric Rating Scale) and systemic metabolism (measured by continuous glucose monitoring).
2) Compare brain fuel utilization measured by PET scan (cerebral uptake of 11C-Acetoacetate + 18 Fluorodeoxyglucose) between AP-naïve patients with a first episode of psychosis and an existing dataset of healthy controls (matched for age and sex).

Exploratory Objectives

Examine associations before and during AP treatment with other measures of psychopathology (e.g., cognition, depressive symptoms, etc.) and metabolism (weight, lipids, inflammation markers, etc.), general systemic impact (renal, hepatic function, inflammation, etc.), and brain structure (MRI).

Compare continuous glucose monitoring data to plasma glucose metabolism measures, as well as the acceptability and feasibility of this monitoring.

5 Study Population

Target Population:
Individuals aged 18 to 35 from the Estrie region who have been evaluated by the PEP team and wish to start an antipsychotic (AP) for the treatment of a first episode of psychosis.

5.1 Sample Size

This is a pilot observational study. Currently, there are no studies that have measured cerebral use of 11C-Acetoacetate before and after AP administration. We must rely on our previous studies on energy metabolism, which showed differences in the same variables with groups of 7 to 15 participants.

Number of participants:18 participants to reach 10 who complete the study. This pilot study will allow us to obtain preliminary data to secure funding for future studies.

5.2 Inclusion Criteria

- Individuals aged 18 to 35.
- Admission to the PEP clinic in Estrie, either outpatient or inpatient, according to the transdiagnostic PEP model.
- Willingness to begin taking an AP (regardless of type and dose, or change in type and dose during the study).
- Ability to read and express themselves in French or English.
- Capable of understanding and signing consent.

5.3 Exclusion Criteria

- Pregnancy, childbirth in the last 6 months, or breastfeeding.
- Presence of a metallic object in the body that is incompatible with MRI.
- Any use of APs for more than 2 continuous weeks in the past year and/or 6 weeks in a lifetime (except for aripiprazole if taken at less than 2.5 mg/day or quetiapine at less than 50 mg/day, regardless of duration or timing of the prescription).
- The following comorbidities: psychosis + borderline or intellectual disability, autism spectrum disorder, substance use disorder with decompensation, psychosis induced by a medical condition, or psychosis induced by drug use or withdrawal.
- Type 1 diabetes.
- Uncontrolled acute suicidal ideation.
- Other conditions that could interfere with participation according to the judgment of the qualified physician.

5.4 Withdrawal Criteria During the Study

The participant's participation in the research may be terminated by the responsible researchers or the participant themselves. This will occur if new discoveries or information indicate that participation in the study is no longer in the participant's best interest, if it becomes apparent that the participant is not following the study's guidelines, or if there are administrative reasons to abandon the study.

Definitive withdrawal from the study will occur in the following cases:

- Voluntary withdrawal. A participant may withdraw their consent to participate in the research at any time without affecting the services and treatments offered.
- Inability to complete initial visits within the 10-day timeframe.
- Initiation of AP medication (other than what is permitted under concomitant medication below) before completing the 2 "BEFORE" visits (depending on the participant's desire or clinical changes).
- Any changes deemed by the responsible physician to be contraindications to continuing in the study (significant renal or hepatic insufficiency, etc.).

5.5 Concomitant Medication

Period before the "V2- Imaging BEFORE" visit

Typically, during the evaluation period at the PEP clinic before the introduction of an antipsychotic at antipsychotic doses, a "non-AP dose" medication may be prescribed to treat certain symptoms (distress, sleep) and will be accepted as part of the study. This includes:

- Quetiapine 12.5 mg to 50 mg PO HS PRN.

An anxiolytic may also be prescribed for imaging:

- Alprazolam 0.25 to 0.5 mg PO BID PRN.

Period between the "V2- Imaging BEFORE" visit and the 2 final visits

Any medication prescribed by the treating team (e.g., to treat side effects, etc.) or taken over the counter by the participant will not result in exclusion but will be documented.

6 Research Methodology

6.1 Study Duration

Total projected duration of the project: 18 months
Recruitment period duration: 16 months
Projected participation duration for participants: Between 5 and 7 weeks

6.2 Recruitment

Participants will be among the newly admitted patients to the PEP program of the Psychiatry Department of CIUSSS de l'Estrie-CHUS. Only patients for whom a psychiatrist deems it appropriate and safe to undergo tests before starting an AP will be invited to participate. After identifying a potentially eligible patient by a psychiatrist, a member of the PEP team or the doctor will ask for permission to be contacted for research participation. Then, a member of the research team (researcher or research professional) will contact the person to present the project and obtain their consent.

6.3 Summary of the study

Table 1. Summary of study procedure	min	Eligibility	PRE	TEP pre	POST	TEP post
Weeks since admission to clinque PEP		*0-1*	*0-1*	*0-1*	*6-10*	*6-10*
Consent	15	X				
Blood test	5		x		x	
Physical measure	10		X		X	
MRI eligibility questionnaire	2	X		X		X
Prenancy test	2	x		X		X
DUP	2	X	X			
Brief Psychiatric Rating Scale (BPRS)	15		X		X	
Clinical Global Impression, Severity (CGI-S)	5		X		X	
Calgary Depression Scale for Schizophrenia (CDSS)	10		X		X	
Global Assessment of Functioning (GAF)	2		X		X	
Brief Assessment of Cognition (BACS)	30		X		X	
Side Effect Rating Scale (UKU)	20		X		X	
Alcohol Use Disorders Identification Test (AUDIT)	2		X		X	
Drug Disorders Identification Test (DUDIT Drug	2		X		X	
Fagerström Test for Nicotine Dependence (FTND)	2		X		X	
Sleepiness Scale (ESS)	1		X		X	
Simple Physical Activity-questionnaire (SIMPAQ)	5		X		X	
Barnes akathysia scale (BAS)	2			X		X
Food Craving questionnaire (FCQ)	5			X		X
Medication list (type + dose)	2	X			X	

Adherence questionnaire (MARS)	2				X	
PET scan	120			X		X
MRI	30			X		
Continuous glucose monitoring	15			X		X

6.4 Study Procedures

After the eligibility visit (V0-eligibility), the participant will be invited to complete the V1-clinical and V2-PET visits before starting the medication ("BEFORE"), but no later than 10 days after the first visit with the psychiatrist.

The two other visits, V3-clinical and V4-PET, will occur 4 to 6 weeks "AFTER" the start of antipsychotic medication.

V0-Eligibility Visit

Location: Hôtel-Dieu
Duration:1 hour

Procedure:
The participant is invited to a brief meeting with a research team member after one of their weekly hospital visits or at their convenience. After presenting the information and consent form and obtaining the signature, various questionnaires will be completed. A snack will be provided.

- Verification of eligibility criteria.
- Sociodemographic data questionnaire.
- Medication list.
- MRI contraindication questionnaire.
- Planning of subsequent visits.

V1 and V3 Clinical Visits (BEFORE and AFTER)

Location: Hôtel-Dieu
Duration:2 hours

Procedure:

- Fasting Blood Draw: 12 hours fasting required.
- Meal/snack provided after blood draw.
- Measurements: Weight, height, waist circumference.
- Blood pressure and pulse.
- Questionnaires will be done as listed in Table 1.
- Breaks will be offered to the participant (snack, coffee).

V2 and V4 Imaging Visits (BEFORE and AFTER)

Location: Centre for Molecular Imaging of Sherbrooke (CHUS Fleurimont)
Duration: 3 hours (fasting for 4 hours before)

Procedure:

- Reconfirm consent.
- Verification of MRI contraindication questionnaire.
- Pregnancy Test
- 15 minutes MRI Scan (volumetric and vascular system imaging).
- Two intravenous catheters will be installed in the forearms, and blood glucose will be measured.
- 20 minutes PET Scan with 11C-Acetoacetate with blood sampling.
- Break: 40 minutes break.
- 30 minutes PET Scan with 18F-FDG with blood sampling.
- Snack provided after the imaging session.

Continuous Glucose Monitoring (Optional)

At the end of the imaging visit, the continuous glucose monitor (Dexcom G7) will be applied, and instructions will be provided (synchronization period, precautions for bathing, etc.). The device will be removed between 8 and 10 days later, according to what suits the participant best.

The compatibility of the device with the participant's mobile phone will be verified, and their agreement will be obtained to install the application.

6.5 Additional Details

Participant Freedom: The participant is free to attend with a companion. For all visits, individualized support by a research team member (preferably the same person) will be provided upon arrival. A taxi will be provided for all participant transportation.

Imaging Appointments: Depending on the availability of imaging slots, but always trying to accommodate the participants as much as possible (availability, fatigue, etc.) and ensuring adherence to protocol timelines, the PET and MRI scans may be separated into two visits.

6.6 Detailed Procedures

Blood Sampling for Metabolism

Blood test - metabolism

- General panel*: Complete blood count, renal function (creatinine), liver function (AST, albumin), thyroid function (TSH), inflammation marker (C-reactive protein).
- Metabolic profile (glucose, insulin, hemoglobin A1c, triglycerides, cholesterol, lactate, insulin, ketones**).
- Metabolomics.
- Cytokines.
- Inflammatory profiles.

*Upon admission to the PEP clinic, a general panel is performed (CBC, TSH, basic liver panel, basic renal panel, sodium, potassium, lipid profile, HbA1c). If the results are already available in the medical record, the panel will not be repeated during the "BEFORE" visit.

**Certain analyses will be conducted in Professor Cunnane's laboratory. Plasma will be frozen for some analyses to be performed at a later time.

MRI Imaging

No specific preparation is required by the participant. Upon arrival, the participant must wear a hospital gown and remove any metallic objects (jewelry, watches, glasses, hearing aids, dental prostheses). It is crucial to remove all metallic objects (buttons, staples, hair clips, zippers).

Procedure:

- The participant will lie on their back on a bed that moves into the MRI machine (MRI3T Ingenia Philips).
- The imaging session will take about 10 minutes (plus installation time), during which the participant must avoid moving their head.
- The MRI machine and head coil used in this study are approved by Health Canada and are routinely used at the CHUS Research Center.
- The participant will be monitored throughout the imaging session, and verbal communication will be possible at all times during the exam.

PET Scan Procedure

Two intravenous catheters will be placed in the forearms, and blood glucose will be measured.

- Approximately 15 minutes before the radiotracer injection, the participant will be encouraged to lie down on the examination gurney or on a gurney in a room adjacent to the examination room.
- CT scan will be performed for a few minutes before the PET scan acquisition begins.
- The radiotracer, adjusted to a volume of 5 to 10 ml of saline (NaCl 0.9%), will be injected through one of the catheters.
  - 11C-Acetoacetate:370 MBq (10 mCi)
  - 18F-FDG:185 MBq (5 mCi)
- The PET scan will last 20 to 30 minutes (depending on the radiotracer).
- Repeated blood samples will be taken during the various PET scan acquisitions (10 ml per PET scan) through one of the catheters.
- Since two radiotracers are used during a single imaging session, there will be a 15 to 30-minute break; the participant will be invited to get up, walk around, and use the restroom.
- After the imaging session, the participant's overall health will be checked, and a snack will be provided.

Blood Sampling During PET

For each PET exam, plasma fractions will be separated by centrifugation, and plasma radioactivity will be immediately counted using a scintillation counter to evaluate the radiotracer's blood kinetics. Plasma will then be used to measure glucose and ketones (for tracer uptake calculations).

Continuous Glucose Monitoring

Glucose levels will be monitored using a continuous glucose monitor (Dexcom G7). This portable device features a small disposable sensor that tests glucose levels every five minutes and sends this information

to a transmitter throughout the day and night. The readings are relayed in real-time to a device that can be read by the patient, caregiver, or healthcare provider, even remotely.

The sensor is worn under the skin (often on the abdomen or arm). The sensor will be placed by the nurse during the metabolic visit, and glucose values will be recorded for about 10 days (8 to 12). The participant will wear a sensor at two points during the project: at the beginning and the end of the study.

6.7 Participant Collaboration

The participant must pay attention to certain instructions while wearing the glucose monitor, such as limiting continuous immersion in water (e.g., bathing, swimming) and charging their smartphone every night.

6.8 Radiotracers Used

The radiotracers are produced and distributed by the Centre for Molecular Imaging of Sherbrooke (CIMS). CIMS has cyclotrons for the production of radiotracers for clinical and research use. This production is done according to good manufacturing practices (GMP). The standard operating procedures for synthesis and quality controls are done in collaboration between the principal investigators, a radiochemist, and a pharmacist. The standard operating procedures or the investigator's brochure can be consulted upon request. In addition, a regulatory affairs officer and a quality control officer are in place to ensure compliance with GMP and monitoring during the study.

18F-FDG:(Gludef® Lantheus Imaging) - Sterile solution, intravenous administration, Health Canada approved for clinical use.
11C-Acetoacetate:Sterile solution, intravenous administration, CHUS has Health Canada approval for research use.

6.9 Participant Withdrawal

When the participant stops or is withdrawn from the study, no further data collection or final visit will occur except to document any adverse events that have occurred since the last visit or follow up with the participant to remove the continuous glucose monitor. All data and samples collected until the participant's withdrawal will be retained per applicable standards.

7 Ethical Considerations

7.1 Risks

Delayed Antipsychotic Administration

According to the guidelines of the National Institute for Health and Care Excellence (NICE Clinical Guideline CG 178) for psychosis and schizophrenia in adults: prevention and management, after the initial evaluation by the psychiatrist, 7 days are typically allowed before the initiation of medication to finalize the diagnosis with the treatment team and discuss treatment options with the patient and their family.

For this research project, the administration of antipsychotic medication (AP) may be delayed by an additional 2-3 days to perform the "BEFORE" imaging. The team will always aim to schedule

appointments within the 7-day period; however, if there are limitations (holidays, participant availability, equipment malfunctions, etc.), these additional 2-3 days will be considered while ensuring that the risk associated with this prescription delay remains minimal.

The risks associated with delaying the prescription of AP will be evaluated on a case-by-case basis in collaboration with the patients according to their clinical condition. Any patient who wishes for immediate AP prescription will receive it and will not be eligible for this research project. The majority of patients request additional time themselves to reflect and research the medications before receiving the prescription. Most request the medication introduction at the next appointment, 2 weeks later, and everything proceeds correctly without side effects, based on clinical experience related to the 2-week delay without AP.

The patient is free to change their mind during this period, either to accelerate the start of AP or to refuse AP altogether. In such cases, the patient will be excluded from the study. Additionally, as provided for in the usual care model of the PEP, the patient will be seen once or twice a week by their primary clinician from the PEP clinic for support and guidance towards non-pharmacological approaches for managing stress and psychotic symptoms. In case of an emergency, the patient is advised to contact their primary clinician. Should a change in health status occur during this period, appropriate medical follow-up will be conducted, and if the need for earlier medication initiation arises, the participant will simply be excluded from the study.

Blood Sampling

The risks associated with blood sampling and the installation of a glucose monitor are the same as those for all standard blood draws, including bleeding, bruising, discomfort, dizziness, pain at the sampling site, and the possibility of infection.

MRI

Magnetic resonance imaging (MRI) is completely painless. It is a radiation-free examination. No particular consequences for humans have been described. The only risks are related to implanted pacemakers, heart valves, or any unreported metallic foreign bodies. A detailed and standardized questionnaire is conducted by the technologist to ensure the absence of metallic bodies. Claustrophobic individuals may feel uncomfortable, experience anxiety, or have a panic attack.

Radiation Exposure

For the entire study, the effective dosimetry received by the participant for the entire imaging process will be a maximum of 11.6 mSv, including a 40% safety factor. These effective doses are below the annual threshold limit of 20 mSv set and authorized by CHUS for the participation of healthy subjects in research projects involving radiation. For more details, see the attached document from the CHUS Radiation Protection Officer (Stéphane Mercure): "Radiation Protection Note and Dose Calculation."

7.2 Benefits

It is possible that the participant may benefit personally from their participation in the research project, but this cannot be guaranteed. This study will contribute to knowledge in psychiatry, particularly metabolic psychiatry.

7.3 Compensation

Participants will receive compensation of CAD 60 for visits V0, V1, and V3, and CAD 100 for visits V2 and V4 (a total of 5 visits, amounting to CAD 380) given in cash at each visit, as well as taxi or parking vouchers for their transportation.

7.4 Information and Consent Form

Before undertaking any procedures directly related to the research project, the participant's consent will be obtained through an information and consent form. This form aims to inform the patient about the study in simple and familiar terms. The participant will have all the necessary time to read it and receive satisfactory answers to their questions. A person will always be available for this purpose. This process aims to obtain free and informed consent. Given the potentially more vulnerable status of participants, their understanding of the study and consent will be validated throughout the project. The accompanying person will also be consulted on this matter.

7.5 Voluntary Participation

The participant is free to participate in the research project and to terminate their participation at any time without any impact on the quality of care and services to which they are entitled, or on relationships with the responsible researchers, their treating physician, or other professionals. In case of withdrawal, the participant's well-being and safety will be ensured by study termination and follow-up procedures according to the circumstances at the time. The disclosure of any new information and modifications to the original protocol that may challenge the participant's decision to continue their participation will be communicated. The responsible researchers may also terminate the participant's participation in the research without their consent if it is in the participant's best interest or if they do not adhere to the project's guidelines.

7.6 Significant Incidental Findings

Following the various tests and examinations in the project, it is possible that significant previously unknown anomalies may be identified that are important for the participant's health. In such a case, the participant will be informed, and one of the project's referring physicians will ensure appropriate medical follow-up. It is possible that they will contact the family doctor or other specialists to ensure the participant is properly taken care of. An incidental finding could exclude the participant from the project.

7.7 Confidentiality

All information collected during this study will be treated with the utmost confidentiality. All collected samples and data will be de-identified. The key will be kept electronically separate from other project data and held by the principal investigator. Dr. Zemmour, the psychiatrist from the PEP team and the project's referring physician, is also the principal investigator of this research project. However, he will only have access to certain anonymized data, notably brain imaging and PET results. Access to information stored on computers is restricted by using a password and firewall. Any data used in future publications will be anonymous so that no data specific to the different study participants will allow them to be identified as individuals.

The consent form clearly mentions the individuals who will or may have access to the information related to this research, namely the principal researchers, associated researchers, their collaborators, and, more rarely, representatives of the research ethics committee. All these individuals are subject to confidentiality rules. Authorization for the reuse of data and biological samples will be requested from participants. Research data may thus be used in other studies on brain metabolism approved by the CIUSSS de l'Estrie - CHUS Research Ethics Board. Therefore, data will be kept by the principal investigators. Given their conservation period, biological samples will be stored for a maximum of 10 years and then destroyed securely.

Participants will be informed that some brain imaging data or "scan images" may be used in scientific publications. The "scan images" obtained after image processing are data reconstructed from algorithms and do not allow re-identification of the persons.

7.8 Storage of Samples and Data

Given their conservation period, de-identified biological samples will be stored at -80°C in a restricted access freezer at the Research Center on Aging (CDRV) for a maximum of 10 years, then destroyed securely according to institutional procedures.

Data collected during the study will be stored on the Research Center on Aging's computer system.

Participants are identified in the computer system by a code. No other personal information that could identify the participants is present, except for the date of birth. Some de-identified paper source documents (questionnaires, blood test results, etc.) will be stored in the participant's file in locked filing cabinets. These cabinets are located in restricted-access offices at the CDRV.

Data will be stored for 25 years. PET scan data will be stored in accordance with Health Canada's policy.

7.9 Insurance

All involved researchers and physicians are covered by professional liability insurance.

7.10 Clinical Trial Registration

To reduce bias and meet publication requirements, this study will be registered in the public registry [www.clinicaltrial.gov](https://clinicaltrial.gov).

7.11 Study Limitations

Several limitations emerge from this pilot study. We have tried to minimize or measure potential confounders and biases within the constraints of a first pilot study.

Diagnostic Variability:
The participants included in the study will likely have different final diagnoses. The PEP clinic model is transdiagnostic, meaning a patient is admitted for a "first episode of psychosis," but the final diagnosis may vary between psychosis NOS (Not Otherwise Specified), chronic psychosis, brief psychotic disorder, schizophreniform disorder, schizophrenia, bipolar disorder, personality disorder, or delusional disorder. The PEP clinic's objective is to provide services to all these patients. We have chosen not to exclude

these sub-pathologies or other comorbidities. This will be a limitation of the study, as the different pathologies may present different metabolic profiles or responses, but we believe that metabolic disturbances are common across these pathologies.

Antipsychotic Type and Dose Variability:
We also chose not to exclude participants based on the type of AP used for treatment or the chosen dose, which may not correspond to the standard effective dose according to the literature, because the PEP clinic's approach is based on clinical evaluation rather than theory, and the study is intended to reflect real-world conditions. Therefore, the determination of this dose is done with the patient and varies for each patient. It is possible that the dose or type (or change) of AP may have a different impact on metabolism. Given the small sample size, it will not be possible to create subgroups. This should be addressed in a future study. A dose equivalency table will be used retrospectively to determine the number of participants with theoretically effective doses, but some variability is possible and would be a limitation of the study.

Adherence to Treatment:
Similarly, adherence to treatment is another concern. We have included an adherence questionnaire to try to control this parameter, but self-reported instruments have their own limitations. Particularly, verifying medication intake is challenging in psychiatric illnesses. Plasma AP levels could be considered in a future study.

Substance Use as a Confounder:
Drug, alcohol, or concomitant medication use is another confounding factor, presenting a limitation to the study. We have planned questionnaires to document it, but self-reported instruments also have limitations.

Nutritional and Lifestyle Factors:
The impact of dietary habits and potential follow-up with a nutritionist or dietitian, or even following a "diet" independently, could introduce variations in the study. It would be interesting to evaluate spontaneous dietary changes in a future study, as they are known to occur with AP use.

Control Group Limitations:
The control group will be drawn from a database of healthy participants obtained in the past 10 years and characterized as metabolically healthy. They may not be representative of the current general population. A control group in a future study would be relevant.

Continuous Glucose Monitoring:
Data obtained by continuous glucose monitoring will likely be more informative than standard plasma measurements, but in this exploratory study, we propose making this part optional to obtain at least preliminary exploratory results and assess the acceptability and feasibility for guidance in a future study.

8 Variables and analysis

8.1 Analyses

All data will be compiled, and differences BEFORE vs. AFTER for the various measurement variables will be assessed using Wilcoxon signed-rank non-parametric tests for paired data.

Correlations will also be performed, particularly between brain function (variables related to global and regional changes in cerebral energy metabolism), blood levels of various measured parameters, and scores from clinical questionnaires (global psychopathology, functioning, depression, cognition, etc.).

In a second phase, comparisons will be made with a control group from a brain imaging database conducted in age-matched participants without the condition (Project 08-111 Cunnane).

The significance threshold will be set at $p < 0.05$. All statistical analyses will be performed using SPSS 24.0 software (SPSS Inc, Chicago, USA).

As this clinical study includes an exploratory component, new analyses or complementary analyses may be added based on the literature or preliminary analyses.

PET Image Reconstruction and Analysis Procedures

The data obtained during the various PET acquisitions will be reconstructed to produce time-based 3D image series. Only reconstruction algorithms approved for use with the device (operation software approved by Health Canada during the scanner's approval process) will be used. PET images will be processed and analyzed using PMOD imaging software with a voxel-based and region-specific analysis method validated by Professor Cunnane's laboratory.

For brain imaging data, a correction for multiple comparisons will be applied (false discovery rate correction).

MRI Image Analysis

MRI acquisitions conducted during the two visits include various modalities to measure, among other things, cerebral perfusion, brain activity, brain volumes, and the integrity of white matter tracts. Image processing and analyses will be conducted in collaboration with collaborator Kevin Whittingstall.

8.2 List of variables

Study variables will be collected by trained and delegated team members using the instruments cited in the protocol or based on laboratory and imaging analyses. Depending on the type of variable (clinical, imaging, etc.), appropriate training will be provided and documented according to SOPs and GCP guidelines by the team physician or expert collaborators. Wherever possible, evaluations for the same participant will be conducted by the same evaluator.

Certain source data (e.g., medication lists, CHUS analysis results, etc.) will be extracted from the electronic medical record system Ariane, but only for data and time points specified in the protocol. Other source data will come from scores obtained from the instruments, laboratory analyses, or imaging analyses.

Data will be entered into a computerized database (Excel file or REDCap depending on the type of data). The procedure will be detailed in the study manual, along with data entry and verification/validation procedures.

Primary variables related to changes in cerebral energy metabolism:

- Cerebral metabolic rate of glucose (µmol/100 g/min) quantified with 18F-FDG;
- Net glucose influx with 18F-FDG (Kglu; min-1);
- Cerebral metabolic rate of acetoacetate with 11C-AcAc (µmol/100 g/min);

- Net acetoacetate influx with 18F-FDG (Kglu; min-1).

Secondary variables related to clinical improvement:

- % change in the Brief Psychiatric Rating Scale (raw score after/raw score before*100).

Secondary variables related to systemic metabolism:

- Plasma glucose concentrations (mM);
- Plasma insulin concentrations (pM);
- Plasma HbA1c concentration (%);
- HOMA-IR score.

Secondary variables related to changes in cerebral energy metabolism in a healthy control group matched by age and sex (Professor Cunnane's database):

- Cerebral metabolic rate of glucose (µmol/100 g/min) quantified with 18F-FDG;
- Net glucose influx with 18F-FDG (Kglu; min-1);
- Cerebral metabolic rate of acetoacetate with 11C-AcAc (µmol/100 g/min);
- Net acetoacetate influx with 18F-FDG (Kglu; min-1).

Exploratory variables related to psychopathology and clinical status:

- DUP;
- Medication lists and dose equivalence;
- Raw scores for various clinical questionnaires:
    - Clinical evaluation and symptoms: BPRS, CGI-S, ESS, BAS;
    - Depression: CDSS;
    - Functioning: GAF;
    - Cognition: BACS;
    - Medication: UKU, MARS;
    - Alcohol and drug consumption: AUDIT, DUDIT, FTND;
    - Physical activity: SIMPAQ.

Exploratory variables related to metabolic and systemic changes:

- Plasma concentrations of total ketones (mM);
- Plasma concentrations of triglycerides, total cholesterol, and free fatty acids (mM);
- Laboratory analysis parameters: Complete blood count, renal function (creatinine), liver function (AST, albumin), thyroid function (TSH), inflammation marker (C-reactive protein);
- Physical measurements (weight, height, waist circumference, blood pressure);
- Metabolomics;
- Cytokine and inflammatory profiles (plasma concentration).

Secondary variables related to structural changes measured by MRI:

- Global and regional brain volumes (ml);
- Cortical thickness (mm);
- Structural changes in brain vasculature (score).

Exploratory variables related to continuous glucose monitoring:

- Average glucose concentration;
- Standard deviation and coefficient of variation;
- Time in range.

9 Data Quality, Safety, and Monitoring

9.1 Good Clinical Practices

The study protocol and the management of the study's quality and safety are based on good clinical practices (GCP) and the institution's standard operating procedures (SOPs). The research staff are or will be trained on these standards before participating in the study.

9.2 Data Management and Validation

A data management procedure, including a system for double entry and data verification, has been established for this project to ensure that the data collected in the study are accurate and complete.

9.3 Quality, Safety, and Ethical Management

Deviations and violations of the protocol will be managed according to the institution's SOPs. The principal investigator is responsible for informing the Research Ethics Board (REB) in case of unplanned changes that could affect the integrity, dignity, or well-being of the participant, or that impact the ethical and scientific aspect of the project. Depending on the situation, corrective measures or a corrective action plan will be implemented.

Additionally, information on participant recruitment, compliance with inclusion/exclusion criteria, the consent process, dropouts, and compliance will be recorded and discussed throughout the study with the principal investigator and the qualified physician.

The goals are to:

- Evaluate whether the risks/benefits for the participant align with what was anticipated.
- Assess whether recruitment and dropout rates correspond to what was anticipated.
- Determine if protocol deviations require changes.
- Ensure that the protocol and ethical processes are respected.

10 Adverse Events Management

Since this project does not involve any interventions, adverse events that may occur during the study could only be related to the radiotracers. Follow-up will be conducted with the PET scan's responsible physician, Dr. Éric Turcotte.

Any adverse event occurring more than 24 hours after the radiotracer injection will not be considered related to the radiotracer or the study. Any side effects related to the APs will not be considered related to the project since they are prescribed with necessary medical follow-up by the treating team at the PEP clinic.

Adverse events will be managed and documented according to the institution's SOPs. Given the specific nature of the radiotracers and the clinical study, the following clarifications are to be considered when managing adverse events involving positron-emitting radiotracers:

- Medical conditions present before the injection of the radiotracers will not be considered adverse events.

- A deterioration of an evolving disease present before the radiotracer injection and not temporally related to the injection will not be considered an adverse event.
- Hospitalization or death due to the initial disease will not be reported as an adverse event.
- Injury related to poor intravenous catheter placement technique will not be considered an adverse event.

In the event of an adverse reaction to a radiotracer (Adverse Drug Reaction, ADR)—i.e., an adverse event for which there is a causal relationship—the CIMS adverse event form will be completed. If the event meets the criteria for rapid reporting—i.e., if there is a causal relationship with the radiotracer, and if it is serious and unexpected—it will also be reported to the CIUSSS de l’Estrie – CHUS REB and Health Canada’s Biologics and Radiopharmaceuticals Therapies Directorate according to current regulations.

11 Potential Impact and Benefits of the Project

In addition to its scientific and medical impacts, this multidisciplinary project fosters unprecedented institutional collaborations between various departments and universities across Canada.
This study will contribute to advancing knowledge in psychiatry, particularly in metabolic psychiatry.

- Potential discovery of the first biomarker for a metabolic issue underlying psychosis, which would be a valuable tool for diagnosing subtle and prodromal forms of the condition. This biomarker could also help engage patients who are more resistant or in denial about their illness, guiding clinicians to initiate care. Additional studies will be necessary to confirm these findings.
- Development of new treatment strategies:
    - Recommendations for prescribing minimally effective doses of antipsychotic medication in monotherapy.
    - Recommendations to combine these low-dose antipsychotic prescriptions with dietary habits low in sugar, which would support the brain and body by reducing the metabolic effects of carbohydrates and insulin resistance.
- Scientific foundation for the development of new treatments targeting ketones (such as exogenous ketone formulations) and cerebral metabolism, similar to how the discovery and production of insulin had a major impact on patients with diabetes.

12 References

1. Goeree, R. *et al.* The economic burden of schizophrenia in Canada in 2004. *Current medical research and opinion* **21**, 2017–28 (2005).
2. Kahn, R. S. *et al.* Schizophrenia. *Nature reviews. Disease primers* **1**, 15067 (2015).
3. Perälä, J. *et al.* Lifetime prevalence of psychotic and bipolar I disorders in a general population. *Archives of general psychiatry* **64**, 19–28 (2007).
4. Kennedy, J. L., Altar, C. A., Taylor, D. L., Degtiar, I. & Hornberger, J. C. The social and economic burden of treatment-resistant schizophrenia: a systematic literature review. *International clinical psychopharmacology* **29**, 63–76 (2014).
5. Drake, R. J. *et al.* Effect of delaying treatment of first-episode psychosis on symptoms and social outcomes: a longitudinal analysis and modelling study. *The lancet. Psychiatry* **7**, 602–610 (2020).
6. Starzer, M. *et al.* 20-year trajectories of positive and negative symptoms after the first psychotic episode in patients with schizophrenia spectrum disorder: results from the OPUS study. *World psychiatry : official journal of the World Psychiatric Association (WPA)* **22**, 424–432 (2023).
7. Harrison, G. *et al.* Recovery from psychotic illness: a 15- and 25-year international follow-up study. *The British journal of psychiatry : the journal of mental science* **178**, 506–17 (2001).
8. Lieberman, J. A. *et al.* Science and recovery in schizophrenia. *Psychiatric services (Washington, D.C.)* **59**, 487–96 (2008).
9. Emsley, R., Nuamah, I., Hough, D. & Gopal, S. Treatment response after relapse in a placebo-controlled maintenance trial in schizophrenia. *Schizophrenia research* **138**, 29–34 (2012).
10. Takeuchi, H. *et al.* Does relapse contribute to treatment resistance? Antipsychotic response in first- vs. second-episode schizophrenia. *Neuropsychopharmacology : official publication of the American College of Neuropsychopharmacology* **44**, 1036–1042 (2019).
11. Andreasen, N. C., Liu, D., Ziebell, S., Vora, A. & Ho, B. C. Relapse duration, treatment intensity, and brain tissue loss in schizophrenia: a prospective longitudinal MRI study. *The American journal of psychiatry* **170**, 609–15 (2013).
12. Owen, M. J., Sawa, A. & Mortensen, P. B. Schizophrenia. *Lancet (London, England)* **388**, 86–97 (2016).
13. van Os, J. & Kapur, S. Schizophrenia. *Lancet (London, England)* **374**, 635–45 (2009).
14. Fan, Z., Wu, Y., Shen, J., Ji, T. & Zhan, R. Schizophrenia and the risk of cardiovascular diseases: a meta-analysis of thirteen cohort studies. *J Psychiatr Res* **47**, 1549–56 (2013).
15. Annamalai, A., Kosir, U. & Tek, C. Prevalence of obesity and diabetes in patients with schizophrenia. *World journal of diabetes* **8**, 390–396 (2017).
16. Correll, C. U. *et al.* Weight Gain and Metabolic Changes in Patients With First-Episode Psychosis or Early-Phase Schizophrenia Treated With Olanzapine: A Meta-Analysis. *The international journal of neuropsychopharmacology* **26**, 451–464 (2023).
17. Sabé, M. *et al.* Comparative Effects of 11 Antipsychotics on Weight Gain and Metabolic Function in Patients With Acute Schizophrenia: A Dose-Response Meta-Analysis. *The Journal of clinical psychiatry* **84**, (2023).
18. Miyakoshi, T. *et al.* Risk factors for abnormal glucose metabolism during antipsychotic treatment: A prospective cohort study. *J Psychiatr Res* **168**, 149–156 (2023).
19. Vancampfort, D. *et al.* Risk of metabolic syndrome and its components in people with schizophrenia and related psychotic disorders, bipolar disorder and major depressive disorder: a systematic review and meta-analysis. *World psychiatry : official journal of the World Psychiatric Association (WPA)* **14**, 339–47 (2015).
20. Smith, E. *et al.* Adiposity in schizophrenia: A systematic review and meta-analysis. *Acta psychiatrica Scandinavica* **144**, 524–536 (2021).

21. Raben, A. T. *et al.* The Complex Relationship between Antipsychotic-Induced Weight Gain and Therapeutic Benefits: A Systematic Review and Implications for Treatment. *Frontiers in neuroscience* **11**, 741 (2017).

22. Smith, E. C. C. *et al.* Clinical improvement in schizophrenia during antipsychotic treatment in relation to changes in glucose parameters: A systematic review. *Psychiatry research* **328**, 115472 (2023).

23. Danan, A., Westman, E. C., Saslow, L. R. & Ede, G. The Ketogenic Diet for Refractory Mental Illness: A Retrospective Analysis of 31 Inpatients. *Frontiers in psychiatry* **13**, 951376 (2022).

24. Sethi, S. *et al.* Ketogenic Diet Intervention on Metabolic and Psychiatric Health in Bipolar and Schizophrenia: A Pilot Trial. *Psychiatry research* **335**, 115866 (2024).

25. Agarwal, S. M. *et al.* Pharmacological interventions for prevention of weight gain in people with schizophrenia. *Cochrane Database Syst Rev* **10**, CD013337 (2022).

26. Lee, J. *et al.* Glucose dysregulation in antipsychotic-naive first-episode psychosis: in silico exploration of gene expression signatures. *Transl Psychiatry* **14**, 19 (2024).

27. Shah, P. *et al.* Alterations in body mass index and waist-to-hip ratio in never and minimally treated patients with psychosis: A systematic review and meta-analysis. *Schizophrenia research* **208**, 420–429 (2019).

28. Henkel, N. D. *et al.* Schizophrenia: a disorder of broken brain bioenergetics. *Molecular psychiatry* **27**, 2393–2404 (2022).

29. Townsend, L. *et al.* Brain glucose metabolism in schizophrenia: a systematic review and meta-analysis of (18)FDG-PET studies in schizophrenia. *Psychological Medicine* **53**, 4880–4897 (2023).

30. Yuksel, C. *et al.* Abnormal Brain Bioenergetics in First-Episode Psychosis. *Schizophrenia bulletin open* **2**, sgaa073 (2021).

31. Andreasen, N. C. *et al.* Hypofrontality in schizophrenia: distributed dysfunctional circuits in neuroleptic-naïve patients. *Lancet (London, England)* **349**, 1730–4 (1997).

32. Agarwal, S. M. *et al.* Brain insulin action in schizophrenia: Something borrowed and something new. *Neuropharmacology* **163**, 107633 (2020).

33. Agarwal, S. M. *et al.* Brain insulin action: Implications for the treatment of schizophrenia. *Neuropharmacology* **168**, 107655 (2020).

34. Guenette, M. D., Chintoh, A., Remington, G. & Hahn, M. Atypical antipsychotic-induced metabolic disturbances in the elderly. *Drugs & aging* **31**, 159–84 (2014).

35. Matéos, M. *et al.* Advanced imaging in first episode psychosis: a systematic review. *Journal of neuroradiology = Journal de neuroradiologie* **50**, 464–469 (2023).

36. Croteau, E. *et al.* [(11)C]-Acetoacetate PET imaging: a potential early marker for cardiac heart failure. *Nucl Med Biol* **41**, 863–70 (2014).

37. Croteau, E. *et al.* Ketogenic Medium Chain Triglycerides Increase Brain Energy Metabolism in Alzheimer's Disease. *Journal of Alzheimer's Disease* **64**, 551–561 (2018).

38. Nugent, S. *et al.* Brain glucose and acetoacetate metabolism: a comparison of young and older adults. *Neurobiol Aging* **35**, 1386–95 (2014).

39. Roy, M. *et al.* A ketogenic supplement improves white matter energy supply and processing speed in mild cognitive impairment. *Alzheimer's & dementia (New York, N. Y.)* **7**, e12217 (2021).

40. Roy, M. *et al.* A ketogenic intervention improves dorsal attention network functional and structural connectivity in mild cognitive impairment. *Neurobiology of aging* **115**, 77–87 (2022).

41. Fortier, M. *et al.* A ketogenic drink improves brain energy and some measures of cognition in mild cognitive impairment. *Alzheimer's & dementia : the journal of the Alzheimer's Association* **15**, 625–634 (2019).

42. Fortier, M. *et al.* A ketogenic drink improves cognition in mild cognitive impairment: Results of a 6-month RCT. *Alzheimers Dement* **17**, 543–552 (2021).

43. Castellano, C. A. *et al.* Regional Brain Glucose Hypometabolism in Young Women with Polycystic Ovary Syndrome: Possible Link to Mild Insulin Resistance. *PLoS One* **10**, e0144116 (2015).

44. Cuenoud, B. *et al.* Cardiorenal ketone metabolism: a positron emission tomography study in healthy humans. *Frontiers in physiology* **14**, 1280191 (2023).
45. Leucht, S. *et al.* Dose equivalents for second-generation antipsychotics: the minimum effective dose method. *Schizophr Bull* **40**, 314–326 (2014).
46. Leucht, S. *et al.* Dose Equivalents for Second-Generation Antipsychotic Drugs: The Classical Mean Dose Method. *Schizophr Bull* **41**, 1397–1402 (2015).
47. Patel, M. X., Arista, I. A., Taylor, M. & Barnes, T. R. E. How to compare doses of different antipsychotics: a systematic review of methods. *Schizophr Res* **149**, 141–148 (2013).
48. Nugent, S. *et al.* Brain and systemic glucose metabolism in the healthy elderly following fish oil supplementation. *Prostaglandins, leukotrienes, and essential fatty acids* **85**, 287–91 (2011).
49. Matthews, D. R. *et al.* Homeostasis model assessment: insulin resistance and beta-cell function from fasting plasma glucose and insulin concentrations in man. *Diabetologia* **28**, 412–9 (1985).
