# Supplementary material for: Brain glucose and ketone metabolism in first-episode psychosis: Neuroimaging and brain metabolism before and after antipsychotic treatment: The protocol for the CAST-ATP study
Source: PLoS One. 2025 Jun 30;20(6):e0325489. doi: 10.1371/journal.pone.0325489 (PMC12208466; doi:10.1371/journal.pone.0325489)
Supplement: S2 File — (DOCX) [file pone.0325489.s002.docx]

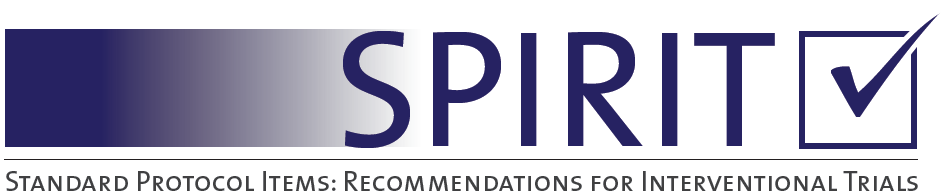


SPIRIT 2013 Checklist: Recommended items to address in a clinical trial protocol and related documents*

| Section/item | ItemNo | Description |
| --- | --- | --- |
| **Administrative information** | | |
| Title | 1 | Brain glucose and ketone metabolism in first-episode psychosis: neuroimaging and brain metabolism before and after antipsychotic treatment: the protocol for the CAST-ATP study |
| Trial registration | 2a | The Sherbrooke site protocol is registered on ClinicalTrials.gov (06651112) |
|  | 2b |  |
| Protocol version | 3 | Original version: V1.3: 2024-09-27 – English translation: 2024-09-27 |
| Funding | 4 | Financial support for this article and the project it describes was received in the form of a philanthropic grant donated by the “Baszucki Brain Research Fund”, USA, to the Université of Sherbrooke Foundation. SCC holds the Endowed Clinical Ketotherapeutics Chair at the Université of Sherbrooke. MH holds the CAMH and UofT Meighen Family Research Chair in Psychosis Prevention. SMA is supported in part by the Discovery Fund, CAMH and the Academic Scholars Awrard from the Department of Psychiatry, University of Toronto. |
| Roles and responsibilities | 5a | K. Zemmour1,2,¶,*, G-O. Samson1,¶, M. Fortier2, E. Parent1, A. Leus3, S. Grignon1, J-D Carrier1, K. Whittingstall4, A. A. Danielsen5,6, O. Köhler-Forsberg5,6, AK Hansen6,7, S.M. Agarwal9,10,11 , A.C. Andreazza 8,9,10,11,12, B. H. Ebdrup13,14, M. Hahn8,9,10,11,14, &, S.C. Cunnane2,15, &  1 Department of Psychiatry, Faculty of Medicine and Health Sciences, University of Sherbrooke, Sherbrooke, Qc, Canada.  2 Research Center on Aging, University of Sherbrooke, Sherbrooke, Qc, Canada.  3 Community members, living with a mental health disorder.  4 Department of Diagnostic Radiology, University of Sherbrooke, Sherbrooke, Qc, Canada.  5 Psychosis Research Unit, Aarhus University Hospital Psychiatry, Aarhus, Denmark  6 Department of Clinical Medicine, Aarhus University, Aarhus, Denmark  7 Department of Nuclear Medicine & PET, Aarhus University Hospital, Aarhus, Denmark  8 Department of Pharmacology & Toxicology, University of Toronto, Toronto, Ontario, Canada.  9 Institute of Medical Sciences, University of Toronto, Toronto, Ontario, Canada  10 Department of Psychiatry, University of Toronto, Toronto, Ontario, Canada  11 Centre for Addiction and Mental Health, Toronto, Ontario, Canada  12Mitochondrial Innovation Initiative, MITO2i, University of Toronto, ON, Canada  13 Center for Neuropsychiatric Schizophrenia Research (CNSR), Mental Health Center, Glostrup, Copenhagen University Hospital – Mental Health Services CPH, Copenhagen, Denmark  14 Department of Clinical Medicine, Faculty of Health and Medical Sciences, University of Copenhagen, Copenhagen, Denmark  15 Division of Endocrinology, Department of Medicine, University of Sherbrooke, Qc, Canada  ^¶^ Sharing co-first authorship  ^&^ Sharing co-senior authorship  Authors contribution: Conceptualization, KZ, MF, AKH, SCC and MH; methodology, KZ, MF, AKH, SCC and MH; writing—original draft preparation, KZ, GOS, EP, BE, MF, SCC; writing—review and editing, KZ, GOS, EP, SG, JDC, AL, MF, AAD, OKF, KW, AKH, BE, MA, AA, SCC and MH; funding acquisition, MH and SC. All authors have read and agreed to the published version of the manuscript. |
|  | 5b | * Correspondence: Email: Margaret.Hahn@camh.ca (MH), Stephen.Cunnane@USherbrooke.ca (SCC) and kevin.zemmour@usherbrooke.ca (KZ) |
|  | 5c |  |
|  | 5d |  |
| Introduction |  |  |
| Background and rationale | 6a | Done in manuscript |
|  | 6b | non |
| Objectives | 7 | The hypotheses are that: (i) lower global and regional glucose uptake will be observed in AP-naïve FEP compared to matched healthy controls, a difference that will be exacerbated 4 to 6 weeks after the initiation of AP, and brain ketone metabolism will not be significantly changed in FEP compared to healthy controls, independently of 4 to 6 weeks post-initiation of AP treatment. (ii) Lower brain glucose uptake will be directly correlated with plasma lactate as an indirect measure of mitochondrial dysfunction, and with impaired peripheral glucose metabolism and cognitive performance before and after AP initiation.  Primary Objective: To compare brain uptake of glucose and ketones between AP-naïve FEP and healthy controls (matched for age and sex), before and 4-6 weeks after AP initiation.  Secondary Objective: In FEP, to compare the measures of brain energy metabolism to clinical improvement over time, as measured by the percentage change in the Brief Psychiatric Rating Scale or Positive and Negative Syndrome Scale (BPRS).  Exploratory Objectives: (i) To examine associations in relation to brain energy metabolism to other measures of psychopathology (e.g., cognition, depressive symptoms), metabolic state (weight, lipids, inflammation markers, systemic glucose metabolism as measured by 10 days of continuous glucose monitoring (CGM)), and brain structure and cerebro-vasculature. (ii) To assess whether CGM data are a valid proxy for brain FDG results. (iii) To assess the acceptability and feasibility of CGM in FEP. |
| Trial design | 8 | Observational study |
| Methods: Participants, interventions, and outcomes | | |
| Study setting | 9 | In the FEP program at CIUSSS de l’Estrie-CHUS (Sherbrooke, Québec) and in the first episode of schizophrenia program (FES, a sub-group of FEP ) of the Central Denmark Region Schizophrenia Cohort (Aarhus, Denmark), participants between 18 and 35 years old, newly admitted to the FEP program, AP-naïve, who wish to start an AP after 10 days of delay, will be invited to participate. |
| Eligibility criteria | 10 | Inclusion criteria  FEP will be diagnosed by a psychiatrist based on the Diagnostic and Statistical Manual of Mental Disorders, 5th ed. (DSM-5-TR) criteria (Sherbrooke), while FES will be diagnosed according to the International Classification of Diseases 10th Revision (ICD-10) criteria (Aarhus). They will be either outpatients or inpatients who are willing to begin taking an AP (regardless of change in drug and/ or dose during the study). Patients will need to be able to read, understand and express themselves in French or English (Sherbrooke), or Danish or English (Aarhus), and must be capable of understanding and signing consent.  Exclusion criteria  Participants will be excluded if they are currently on an AP (see exceptions below), or if there is an history of AP use for morethan 12 weeks or more in their lifetime, with a current wash-out period of 2 weeks, prior to study start, with the exception of injectables, which are excluded. The exception will be aripiprazole if it taken at less than 2.5 mg/day or quetiapine at less than 50 mg/day, regardless of duration or timing of the prescription. These two exclusions will help relieve symptoms of anxiety, distress and insomnia (and, hence, recruitment) with a minimal predicted impact on brain energy metabolism. The following comorbidities will lead to exclusion on both sites: known intellectual disability, autism spectrum disorder, moderate to severe substance use disorder, psychosis induced by a medical condition, psychosis induced by drug use or withdrawal, acute suicidal ideation, diabetes mellitus, and other conditions that could interfere with participation according to the judgment of the qualified physician. These criteria are assessed by the psychiatrist according to review of the patient’s file, the psychiatric interview, and during the first eligibility visit by a member of the research team. Presence of a metallic object in the body that is incompatible with MRI, or pregnancy, childbirth in the last 6 months or breastfeeding that is incompatible with radiotracer injection will also result in exclusion. |
| Interventions | 11a | Only FEP patients for whom the participating psychiatrist deems it appropriate and safe to undergo tests requiring a ≤10-day delay before starting an AP will be invited to participate. A member of the research team (researcher or research professional) will then contact the person to present the project and obtain their written consent. After the first visit to the FEP clinic, patients whom a psychiatrist deems appropriate and safe to participate will be invited to the V0-Eligibility visit, at which time the participant will be informed about the study. Informed consent, eligibility criteria, sociodemographic data, medication list, MRI contraindication, and the subsequent visits are done during this visit (Fig 1). During V1- and V3- Clinic visits (before and after AP), the research nurse will obtain a 12 h fasting blood sample then will measure anthropometrics and vital signs. Face-to-face interviews and self-reported questionnaires with the research team will be completed (Fig 2). Before the V2- and V4- Imaging visits (before and after AP) the participant will need to have fasted for at least 4 hours. The research team will reconfirm consent and complete questionnaires on akathisia symptoms and food craving (Figure 2). A pregnancy test will be repeated for women. The Imaging visits involve MRI and PET scans. Optional CGM (Dexcom G7) will be proposed right after the Imaging visits. Compatibility of the CGM device with the participant’s mobile phone (to be supplied if necessary) will be verified to upload the data and instructions provided (synchronization period, precautions for bathing, etc.). The CGM device will be removed 8-10 days later. |
|  | 11b | Criteria for discontinuing: The patient is free to change its mind during this period, either to accelerate the start of AP or to refuse AP altogether. In such cases, the patient will be excluded from the study. |
|  | 11c | Strategies to improve adherence to intervention protocols: none |
|  | 11d | As provided for in the usual care model of the PEP, the patient will be seen once or twice a week by their primary clinician from the PEP clinic for support and guidance towards nonpharmacological approaches for managing stress and psychotic symptoms. In case of an emergency, the patient is advised to contact their primary clinician. Should a change in health status occur during this period, appropriate medical follow-up will be conducted, and if the need for earlier medication initiation arises, the participant will simply be excluded from the study. |
| Outcomes | 12 | Data will be collected (Fig 2) during face-to-face interviews and be performed by research team members in psychiatric outpatient’s or research center’s offices depending on the most convenient option for participants. Participants will be invited to complete self-reported questionnaires independently or to ask for assistance for all or part of the questionnaires.  Brain ketone and glucose metabolism:  PET scanner, MRI procedure and image analysis:  The Sherbrooke site will perform all PET scans on a Biograph Vision 600 scanner (Siemens, Erlangen, Germany) with a 26-cm axial field of view. The image-derived input function will be cross-calibrated against the plasma radioactivity counted in a gamma counter (Cobra, Packard, USA) of blood samples acquired during each PET session (Castellano 2017, Croteau 2018). [11C]AcAc is used to visualize brain ketone uptake and the site has expertise in multi-imaging approaches, combining multi-tracer PET with various MRI modalities (volumetric, functional, diffusion, etc.) [17, 36, 37, 38]. FEP data will be compared to an existing database of healthy controls from Sherbrooke.  PET scans will be matched to brain regions by an MRI acquired on the same day (3 Tesla with a 32-channel head coil, Ingenia, Philips Healthcare, Best, The Netherlands). Global and regional brain volumes, and thicknesses of the cerebral cortex will be measured. To visualize the cerebral arteries, specifically the lumen diameter and tortuosity of all major intracranial arteries, we will acquire a high resolution, whole-brain multi-band time of flight (TOF) sequence (FOV=200X200X120.9mm, TR/TE=23/3.45ms, FA= 18°, parallel imaging (SENSE) acceleration factor=3, acquisition resolution of 0.65x0.65x1.30mm, reconstructed resolution of 0.626x0.625x0.65mm). This will be followed by a single slice 2D-phase contrast velocity image that was placed using the offset of the labelling band to estimate velocity where the blood is labelled (FOV= 230x230mm; TR/TE=9.2/6; slice thickness=5mm, voxel size= 0.45x0.45mm; bandwidth=428.3Hz/pixel; velocity encoding gradient=80cm/s). For structural T1-weighted images, the protocol is as follows: scan duration = 6 min, repetition time (TR) = 7.9 ms, echo time (TE) = 3.5 ms, field of view= 240×240×150, flip angle = 8 and 1mm3 isotropic voxels. The hypothalamus and pituitary gland volumes will be included because they are brain regions linked with the hypothalamus-pituitary-adrenal (HPA) stress axis, which could be hyperactive in FEP [40], [41], [42].  The Aarhus site will scan on a GE HealthCare SIGNATM PET/MR AIRTM, with the tracers, [15O]H2O, [11C]OHB and [18F]FDG each with simultaneous PET recording of the brain in list mode, automatic drawing of arterial blood samples, and acquisition of MR. MR sequences will include T1, T2 FLAIR and TOF images, and gamma-aminobutyric acid spectroscopy. |
| Participant timeline | 13 | Psychiatric outcome measures:  The history of psychiatric diagnosis and the duration of untreated illness (calculated by subtracting the age at which the first symptoms of psychosis is reported from the age at first meeting with the FEP team) will be assessed by the study psychiatrist based on DSM-V-TR or ICD-10 criteria. Validated questionnaires will be used including the Brief Psychiatric Rating Scale to assess psychotic symptoms [43] in Sherbrooke and the Positive and Negative Syndrome Scale [44] in Aarhus, the Calgary Depression Scale for Schizophrenia for depressive symptoms [45], the Brief Cognitive Assessment in Schizophrenia [46], the Clinical Global Impressions Severity Scale for global severity of symptoms [47], the Global Assessment of Functioning [48], and the Alcohol Use Disorders Identification Test and Drug Use Disorders Identification Test, and Fagerström Test for drug, alcohol and nicotine dependences [49]. Sleep disorder will be assessed by the Epsworth Sleepiness Scale (ESS). Side effects of AP will be measured by the (i) Side Effect Rating Scale (SERS-UKU), and the Barnes Akathysia Scale (BAS). The dose of AP will be adjusted with chlorpromazine equivalents [50]. Adherence to AP will be assessed with the Medication Adherence Rating Scale [51]. Exercise will be measured as average weekly hours of sport/exercise per day as measured by question 4 of the Simple Physical Activity questionnaire SIMPAQ (hour). Medication type and dose will be tracked.  Clinical and laboratory measures:  Anthropometric biomarkers:  Weight, height, body mass index (BMI), waist circumference, blood pressure and pulse will be measured at V1 and V3.  General, metabolic and inflammation biomarkers  The general medical assessment will include a complete blood count, renal function (creatinine), liver function (AST, albumin), thyroid function (TSH), and pregnancy test (β-HCG) for women. The plasma metabolic profile will include fasting glucose, insulin, and hemoglobin A1c, triglycerides, cholesterol, lactate, and ketones, plus metabolomic and inflammatory profiles. Plasma caprylic and capric acids will be measured by gas chromatography-mass spectrometry (GC-MS; Agilent, Waldbronn, Germany). Glucose levels will be monitored from CGM as mean ± SD and time in range. Inflammatory biomarkers will include C-reactive protein, interleukins-6 and 2, and tumor necrosis factor-alpha.  Participant recruitment began in 04 October 2024 and is expected to be completed by 04 February 2026. Considering the additional two months planned for patient participation, data collection and analysis should be finalized by spring 2026.) |
| Sample size | 14 | We are not aware of any existing work measuring brain ketone metabolism before or after AP which we could be used to estimate an appropriate sample size in this population. Our previous studies on brain ketone and FDG PET showed group differences with sample sizes of 7 to 15 participants. These previous results support recruitment of a total of 36 participants with FEP (18 per site). |
| Recruitment | 15 | Only FEP patients for whom the participating psychiatrist deems it appropriate and safe to undergo tests requiring a ≤10 day delay before starting an AP will be invited to participate. A member of the research team (researcher or research professional) will then contact the person to present the project and obtain their written consent. |
| **Methods: Assignment of interventions (for controlled trials)** | | |
| Allocation: |  |  |
| Sequence generation | 16a |  |
| Allocation concealment mechanism | 16b |  |
| Implementation | 16c |  |
| Blinding (masking) | 17a |  |
|  | 17b |  |
| **Methods: Data collection, management, and analysis** | | |
| Data collection methods | 18a | Study variables will be collected by trained and delegated team members using the instruments cited in the protocol or based on laboratory and imaging analyses. Depending on the type of variable (clinical, imaging, etc.), appropriate training will be provided and documented according to SOPs and GCP guidelines by the team physician or expert collaborators. Wherever possible, evaluations for the same participant will be conducted by the same evaluator.  Certain source data (e.g., medication lists, CHUS analysis results, etc.) will be extracted from the electronic medical record system Ariane, but only for data and time points specified in the protocol. Other source data will come from scores obtained from the instruments, laboratory analyses, or imaging analyses.  Data will be entered into a computerized database (Excel file or REDCap depending on the type of data). The procedure will be detailed in the study manual, along with data entry and verification/validation procedures.  Primary variables related to changes in cerebral energy metabolism:  • Cerebral metabolic rate of glucose (μmol/100 g/min) quantified with 18F-FDG; • Net glucose influx with 18F-FDG (Kglu; min-1); • Cerebral metabolic rate of acetoacetate with 11C-AcAc (μmol/100 g/min); • Net acetoacetate influx with 18F-FDG (Kglu; min-1).  Secondary variables related to clinical improvement:  • % change in the Brief Psychiatric Rating Scale (raw score after/raw score before*100).  Secondary variables related to systemic metabolism:  • Plasma glucose concentrations (mM); • Plasma insulin concentrations (pM); • Plasma HbA1c concentration (%); • HOMA-IR score.  Secondary variables related to changes in cerebral energy metabolism in a healthy control group matched by age and sex (Professor Cunnane's database): • Cerebral metabolic rate of glucose (μmol/100 g/min) quantified with 18F-FDG; • Net glucose influx with 18F-FDG (Kglu; min-1); • Cerebral metabolic rate of acetoacetate with 11C-AcAc (μmol/100 g/min); • Net acetoacetate influx with 18F-FDG (Kglu; min-1).  Exploratory variables related to psychopathology and clinical status:  • DUP; • Medication lists and dose equivalence; • Raw scores for various clinical questionnaires: o Clinical evaluation and symptoms: BPRS, CGI-S, ESS, BAS; o Depression: CDSS; o Functioning: GAF; o Cognition: BACS; o Medication: UKU, MARS; o Alcohol and drug consumption: AUDIT, DUDIT, FTND; o Physical activity: SIMPAQ.  Exploratory variables related to metabolic and systemic changes:  • Plasma concentrations of total ketones (mM); • Plasma concentrations of triglycerides, total cholesterol, and free fatty acids (mM); • Laboratory analysis parameters: Complete blood count, renal function (creatinine), liver function (AST, albumin), thyroid function (TSH), inflammation marker (C-reactive protein); • Physical measurements (weight, height, waist circumference, blood pressure); • Metabolomics; • Cytokine and inflammatory profiles (plasma concentration). |
|  | 18b | Secondary variables related to structural changes measured by MRI:  • Global and regional brain volumes (ml);  • Cortical thickness (mm);  • Structural changes in brain vasculature (score).  Exploratory variables related to continuous glucose monitoring:  • Average glucose concentration;  • Standard deviation and coefficient of variation;  • Time in range.  The study protocol and the management of the study’s quality and safety are based on good clinical practices (GCP) and the institution’s standard operating procedures (SOPs). The research staff are or will be trained on these standards before participating in the study.  A data management procedure, including a system for double entry and data verification, has been established for this project to ensure that the data collected in the study are accurate and complete. |
| Data management | 19 | A data management procedure, including a system for double entry and data verification, has been established for this project to ensure that the data collected in the study are accurate and complete. |
| Statistical methods | 20a | PET data analysis:  PET tracer kinetics will be assessed by the Patlak method to quantify the brain uptake rate constants (Kglu, KAcAc [Sherbrooke]¸or KOHB [Aarhus]; min−1) and cerebral metabolic rate (CMRAcAc/OHB; CMRGlu; μmol/100 g/min) [52].  MRI data analysis  Regional and whole brain volumes and cortical thicknesses will be determined using FreeSurfer Suite 6.0 or newer (Martinos Center for Biomedical Imaging, Cambridge, MA) [17]. Regional volumes will be normalized to the intracranial volume of each participant [53]. |
|  | 20b | At both sites, pre-post AP differences will be evaluated using Wilcoxon signed-rank non-parametric tests for paired data. Correlations will also be assessed between brain function (variables related to global and regional changes in brain energy metabolism) and blood levels of the various markers, as well as scores from different clinical questionnaires (global psychopathology, functioning, depression, and cognition). Data will be presented as mean ±SD. All statistical analyses will be performed using SPSS 25.0 software (SPSS Inc., Chicago, Illinois, USA) using two-sided tests with statistical significance defined as p<0.05. Each site will independently compile and analyze scans and outcomes. In an additional exploratory step, federated learning will be used to further analyze and compare the two datasets in greater depth. Federated learning allows analysis of multiple datasets located on different devices, without exchanging and sharing raw data [54]. |
|  | 20c | none |
| **Methods: Monitoring** | | |
| Data monitoring | 21a | DMC is not needed, no competing interest, no obligation toward funding |
|  | 21b | It’s an observational study |
| Harms | 22 | Following the various tests and examinations in the project, it is possible that significant previously unknown anomalies may be identified that are important for the participant's health. In such a case, the participant will be informed, and one of the project's referring physicians will ensure appropriate  medical follow-up. It is possible that they will contact the family doctor or other specialists to ensure the participant is properly taken care of. An incidental finding could exclude the participant from the project.  Since this project does not involve any interventions, adverse events that may occur during the study could only be related to the radiotracers. Follow-up will be conducted with the PET scan’s responsible physician, Dr. Éric Turcotte.  Any adverse event occurring more than 24 hours after the radiotracer injection will not be considered related to the radiotracer or the study. Any side effects related to the APs will not be considered related  to the project since they are prescribed with necessary medical follow-up by the treating team at the PEP clinic.  Adverse events will be managed and documented according to the institution's SOPs. Given the specific nature of the radiotracers and the clinical study, the following clarifications are to be considered when managing adverse events involving positron-emitting radiotracers:  • Medical conditions present before the injection of the radiotracers will not be considered adverse events.  • A deterioration of an evolving disease present before the radiotracer injection and not temporally related to the injection will not be considered an adverse event.  • Hospitalization or death due to the initial disease will not be reported as an adverse event.  • Injury related to poor intravenous catheter placement technique will not be considered an adverse event.  In the event of an adverse reaction to a radiotracer (Adverse Drug Reaction, ADR)—i.e., an adverse event for which there is a causal relationship—the CIMS adverse event form will be completed. If the event meets the criteria for rapid reporting—i.e., if there is a causal relationship with the radiotracer, and if it is serious and unexpected—it will also be reported to the CIUSSS de l’Estrie – CHUS REB and Health Canada’s Biologics and Radiopharmaceuticals Therapies Directorate according to current regulations. |
| Auditing | 23 | No auditing |
| Ethics and dissemination | | |
| Research ethics approval | 24 | Protocol REC CIUSSS de l’Estrie-CHUS: 2025-5589  The study protocol and the management of the study’s quality and safety are based on good clinical practices (GCP) and the institution’s standard operating procedures (SOPs). The research staff are or will be trained on these standards before participating in the study. A data management procedure, including a system for double entry and data verification, has been established for this project to ensure that the data collected in the study are accurate and complete. |
| Protocol amendments | 25 | Deviations and violations of the protocol will be managed according to the institution's SOPs. The principal investigator is responsible for informing the Research Ethics Board (REB) in case of unplanned changes that could affect the integrity, dignity, or well-being of the participant, or that impact the ethical and scientific aspect of the project. Depending on the situation, corrective measures or a corrective action plan will be implemented. Additionally, information on participant recruitment, compliance with inclusion/exclusion criteria, the consent process, dropouts, and compliance will be recorded and discussed throughout the study with the principal investigator and the qualified physician.  The goals are to:  • Evaluate whether the risks/benefits for the participant align with what was anticipated.  • Assess whether recruitment and dropout rates correspond to what was anticipated.  • Determine if protocol deviations require changes.  • Ensure that the protocol and ethical processes are respected. |
| Consent or assent | 26a | Before undertaking any procedures directly related to the research project, the participant's consent will be obtained through an information and consent form. This form aims to inform the patient about the study in simple and familiar terms. The participant will have all the necessary time to read it and receive satisfactory answers to their questions. A person will always be available for this purpose. This process aims to obtain free and informed consent. Given the potentially more vulnerable status of participants, their understanding of the study and consent will be validated throughout the project. The  accompanying person will also be consulted on this matter.  The participant is free to participate in the research project and to terminate their participation at any time without any impact on the quality of care and services to which they are entitled, or on relationships with the responsible researchers, their treating physician, or other professionals. In case of withdrawal, the participant's well-being and safety will be ensured by study termination and follow-up procedures according to the circumstances at the time. The disclosure of any new information and modifications to the original protocol that may challenge the participant's decision to continue their participation will be communicated. The responsible researchers may also terminate the participant's participation in the research without their consent if it is in the participant's best interest or if they do not adhere to the project's guidelines. |
|  | 26b |  |
| Confidentiality | 27 | All information collected during this study will be treated with the most confidentiality. All collected samples and data will be de-identified. The key will be kept electronically separate from other project data and held by the principal investigator. Dr. Zemmour, the psychiatrist from the PEP team and the project's referring physician, is also the principal investigator of this research project. However, he will  only have access to certain anonymized data, notably brain imaging and PET results. Access to information stored on computers is restricted by using a password and firewall. Any data used in future  publications will be anonymous so that no data specific to the different study participants will allow them to be identified as individuals.  The consent form clearly mentions the individuals who will or may have access to the information related to this research, namely the principal researchers, associated researchers, their collaborators, and, more rarely, representatives of the research ethics committee. All these individuals are subject to confidentiality rules. Authorization for the reuse of data and biological samples will be requested from participants. Research data may thus be used in other studies on brain metabolism approved by the CIUSSS de l’Estrie - CHUS Research Ethics Board. Therefore, data will be kept by the principal investigators. Given their conservation period, biological samples will be stored for a maximum of 10 years and then destroyed securely. Participants will be informed that some brain imaging data or "scan images" may be used in scientific  publications. The "scan images" obtained after image processing are data reconstructed from algorithms and do not allow re-identification of the persons. |
| Declaration of interests | 28 | SCC has received research funding and test materials from Nestlé Health Science. He consults for Nestlé Health Science and Cerecin. OKF reports speaker fees from Lundbeck Pharma A/S, consultant work for WCG International, and serving on an advisory board for Boehringer-Ingelheim. BE is part of the Advisory Board of Boehringer Ingelheim, Lundbeck Pharma A/S; and has received lecture fees from Boehringer Ingelheim, Otsuka Pharma Scandinavia AB, and Lundbeck Pharma A/S. SMA has received honoraria from HLS therapeutics and Boehringer Ingelheim. MH has received consultant fees from Alkermes and MERCK. The other authors declare that the research will be conducted in the absence of any commercial or financial relationships that could be construed as a potential conflict of interest. |
| Access to data | 29 | Given their conservation period, de-identified biological samples will be stored at -80°C in a restricted access freezer at the Research Center on Aging (CDRV) for a maximum of 10 years, then destroyed  securely according to institutional procedures. Data collected during the study will be stored on the Research Center on Aging's computer system. Participants are identified in the computer system by a code. No other personal information that could identify the participants is present, except for the date of birth. Some de-identified paper source  documents (questionnaires, blood test results, etc.) will be stored in the participant's file in locked filing cabinets. These cabinets are located in restricted-access offices at the CDRV. Data will be stored for 25 years. PET scan data will be stored in accordance with Health Canada's policy. |
| Ancillary and post-trial care | 30 | All involved researchers and physicians are covered by professional liability insurance. |
| Dissemination policy | 31a | none |
|  | 31b |  |
|  | 31c | none |
| Appendices |  |  |
| Informed consent materials | 32 | Uploaded in “other”. |
| Biological specimens | 33 | Plans for collection, laboratory evaluation, and storage of biological specimens for genetic or molecular analysis in the current trial and for future use in ancillary studies, if applicable |

*It is strongly recommended that this checklist be read in conjunction with the SPIRIT 2013 Explanation & Elaboration for important clarification on the items. Amendments to the protocol should be tracked and dated. The SPIRIT checklist is copyrighted by the SPIRIT Group under the Creative Commons “[Attribution-NonCommercial-NoDerivs 3.0 Unported](http://www.creativecommons.org/licenses/by-nc-nd/3.0/)” license.
