# Supplementary material for: Brain glucose and ketone metabolism in first-episode psychosis: Neuroimaging and brain metabolism before and after antipsychotic treatment: The protocol for the CAST-ATP study
Source: PLoS One. 2025 Jun 30;20(6):e0325489. doi: 10.1371/journal.pone.0325489 (PMC12208466; doi:10.1371/journal.pone.0325489)
Supplement: Attachment — Submitted filename: Response to reviewers_Zemmour et al 20250502.docx [file pone.0325489.s004.docx]

**Response to reviewers- *Zemmour et al., Manuscript 2025***

**Comments to the Author**

**Reviewer #1:**

The objectives (primary, secondary and exploratory) are outlined appropriately and the document is well written. There are a number of clarifications needed as noted below.

1) As per the investigators, this study is observational and will thus have no direct influence on clinical practice. It is expected that a total of n=24 participants will be needed to complete the study on the two sites combined. The investigators note this should provide ample power to define the metabolic perturbations in the brain. How is power being used here?

*RESPONSE: “Ample power” was not meant to indicate a formal power calculation, but rather to reflect our expectation that a sample of 24 is expected be sufficient to detect a meaningful difference in global and regional brain ketone uptake versus brain glucose uptake in FEP. Given that this patient population is totally new in terms of brain glucose and ketone PET imaging, this study is powered to identify clear signals to guide future research.*

2) There are many analyses being proposed for so few patients. One needs to exercise caution on how to interpret the p-values as this is noted as a proof of concept study as per the investigators. This should be noted as a limitation.

*RESPONSE: We agree that, given the small sample size and exploratory nature of the study, p-values must be interpreted with caution. This will be clearly acknowledged as a limitation (change made on page 9).*

3) The sample size rationale appears reasonable as there is no background data to establish it with any power. However, could some clinically meaningful effect sizes for differences be used to establish the sample size? In any case, be specific. If you need 24 subjects from the two sites is that 12 per group (AP vs. controls)?

*RESPONSE: The sample size estimate is based on brain glucose and ketone uptake values obtained from over 400 PET scans across a wide range of ages, clinical conditions, and interventions. While no study to date has investigated this specific population—individuals with a first episode of psychosis (FEP)—using this metabolic imaging approach, our study is exploratory in nature and aims to provide foundational data to guide future, larger trials. Given the expected heterogeneity among FEP participants, we are confident that a sample size of n=24 across both sites (approximately 12 per group) will be sufficient to establish feasibility and detect meaningful patterns in brain fuel metabolism that may inform future effect size estimations and study designs.*

4) Also what about dropouts? How will they be handled in this short period if that should be an issue? This should be added as a possible limitation if such is the case.

*RESPONSE: Analyses will be based on complete data (before and after AP use). In the event of higher-than-expected dropout, we will assess the characteristics of participants lost to follow-up and may explore additional sensitivity or descriptive analyses as appropriate. Also, we have already accounted for potential important attrition by planning to recruit up to 18 FEP participants per site with the goal of obtaining complete data on at least 10 of them. However, if attrition exceeds expectations, it may further reduce statistical power and introduce selection bias, particularly if dropouts differ systematically from completer This will be added as a limitation (changes made page 9).*

5) The statistical analysis approach for this limited sample is reasonable. However, if the investigators are proposing a non parametric approach such as the Wilcoxon signed rank for paired data then one may wish to consider the IQR (interquartile range) and not only the mean and standard deviation for summary results if some data satisfies the normality assumption.

*RESPONSE: Given our small sample size, we agree that a more comprehensive analytical approach could be develop.* *We will assess normality for each variable and choose parametric or non-parametric tests accordingly. Summary statistics will be adapted to the data distribution, using means/SD or medians/IQR as appropriate (change made page 8).*

6) There are a number of outcome measures. Specific analyses should be matched with those being proposed. The timeline in Table 1 was very helpful

RESPONSE: We have revised the manuscript to ensure consistency between the proposed analyses and the corresponding outcome measures (page 8).

Reviewer #2:

1) The protocol’s hypothesis that antipsychotic treatment may worsen brain glucose metabolism, while leaving ketone metabolism intact, is particularly relevant to advancing therapeutic strategies. Another strength is that this protocol is novel in that direct neuroimaging measurement of both glucose and ketone metabolism before and after antipsychotic initiation in FEB is, I believe as you stated, unprecedented. You have clearly referenced recent metabolic psychiatry and ketogenic diet studies related to serious mental illnesses (Lines 77-89), briefly provided examples of existing PET studies (Lines 94-96) and explicitly mentioned the absence of direct measurement of brain glucose and ketone metabolism in FEP (Lines 90-92). A possible improvement would be to add a short paragraph summarizing recent PET imaging or relevant metabolic intervention studies in psychosis if they exist or are underway in clinical trials. This would strengthen the claim making the claim of novelty explicit, verifiable and contextualized clearly within existing research, but only as manuscript length allows. What has already been provided in the manuscript is sufficient. This could be reserved for subsequent publications reporting empirical results from this protocol.

*RESPONSE: We are confident in the statement that no measurements of brain ketone metabolism (PET or MRS) have been reported. We agree that summarizing these emerging studies and relevant metabolic interventions in psychosis could further contextualize our protocol and support the claim of novelty. However, given current manuscript length constraints, we will reserve this for future publications reporting empirical findings from this study.*

2) I appreciate that one of the exclusion criteria is “psychosis induced by drug use or withdrawal” and I like that you state clearly a psychiatrist will make that determination through review of patient file, psychiatric interview and first eligibility visit by a researcher team member. I think that this may still be difficult to determine. Will any standardized structured interviews or special clinical instruments be used to help determine this? Will you rely on or conduct toxicology screenings to rule out recent drug use. How will it be determined in cases of chronic drug use leading up to first psychosis? It may help other researchers using this protocol to have that level of detail from your work.

*RESPONSE: Participants will be patients of our two FEP clinics. “Simple, short” psychosis induced by drug use or withdrawal is an exclusion criterion. However, in the case of chronic use of drug, psychotics symptoms can be longer and may mimic primary psychosis. In that case, where it’s unclear if psychotics symptoms are primary or secondary, such patients may end up being included in this study. Comorbidities will be written up for these participants. This will be noted as a limitation when the results are submitted for publication.*

3) The Aarhus site explicitly mentions having access to MRS to measure GABA. Given the significant metabolic relevance of glutamate, and emerging evidence suggesting substantial glutamatergic reductions with other metabolic interventions (e.g., ketogenic diets), I suggest considering measuring brain glutamate levels via MRS at sites with available equipment. Or possibly peripheral biomarkers reflecting glutamatergic metabolism could also be explored, if feasible. Including glutamate measurements would allow valuable comparison with metabolic changes seen in other interventions and disorders, potentially clarifying how antipsychotic initiation uniquely affects glutamate metabolism in first-episode psychosis. I do recognize, however, that funding or logistical constraints may not allow for such an expansion; I suggest this merely because it could provide interesting insights.

*RESPONSE: This is a great idea and will be explored in a future study.*

4) In regards to secondary and exploratory objectives, these are well aligned. I wonder if the use of a standardized quality of life measure as used in other metabolic psychiatry studies would be well-aligned and enrich the interpretation of the primary outcomes.

*RESPONSE: This is a very relevant and interesting suggestion which we will certainly keep in mind for a future interventional study in development.*

5) Given that sleep disturbances are both common in first episode psychosis and frequently affected (either positively or negatively) by antipsychotic treatment, sleep disruption could represent a confounding variable as you are studying metabolism in this population. I am wondering if explicitly clarifying how you will evaluate this in your statistical analysis plan would strengthen the protocol. Or a mention of whether or how you intend to account analytically for variations in sleep disruption (as measured by the Epworth Sleepiness Scale).

*RESPONSE: Given our small sample size and insufficient statistical power for objective sleep measures, we will still descriptively report individual sleep scores, and we will explore potential correlations with metabolic outcomes in an exploratory manner. The statistical plan was revised to clarify our intend (page 8).*

6) Wilcoxon is clearly justified in the text and appropriate given the sample size and repeated-measures design. Correlational analysis is appropriate, straightforward and adequately described. I particularly like the proposed use of Federated Learning (as described in lines 287-289) as an ethical and statistical choice in studying this vulnerable population across international sites.
7) Overall this protocol is very well done, methodologically sound, extremely clean and professional. That said, I found just a few minor punctuation and formatting points.

   Line 95: "… aripiprazole and ziprasidone are reported be associated …" Correction: should be "are reported to be associated" (missing the word "to").
   Lines 164-165: "The exception will be aripiprazole if it taken at less than 2.5 mg/day…" Correction: should be "if it is taken…" (missing the word "is").
   Line 249: "Side effects of AP will be measured by the (i) Side Effect Rating Scale (SERS-UKU), and the Barnes Akathysia Scale (BAS)." Correction: No comma needed before "and" here since only two items are listed. Better phrasing: "...measured by the Side Effect Rating Scale (SERS-UKU) and the Barnes Akathisia Scale (BAS)."
   Line 357: "Academic Scholars Awrard" Correction: should be "Academic Scholars Award" (typo).

RESPONSE: We have made the necessary corrections to spelling and punctuation errors."

**Additional requirement**

1. Please ensure that your manuscript meets PLOS ONE's style requirements, including those for file naming.

 RESPONSE: We have ensured that the manuscript and all files comply with PLOS ONE’s style requirements.

 2. PLOS requires an ORCID iD for the corresponding author in Editorial Manager on papers submitted after December 6th, 2016. Please ensure that you have an ORCID iD and that it is validated in Editorial Manager. To do this, go to ‘Update my Information’ (in the upper left-hand corner of the main menu), and click on the Fetch/Validate link next to the ORCID field. This will take you to the ORCID site and allow you to create a new iD or authenticate a pre-existing iD in Editorial Manager.

 RESPONSE: ORCID iD was add for the corresponding author.

3. Thank you for stating the following financial disclosure:
Financial support for this article and the project it describes was received in the form of a philanthropic grant donated by the “Baszucki Brain Research Fund”, USA, to the Université of Sherbrooke Foundation. SCC holds the Endowed Clinical Ketotherapeutics Chair at the Université of Sherbrooke. MH holds the CAMH and UofT Meighen Family Research Chair in Psychosis Prevention. SMA is supported in part by the Discovery Fund, CAMH and the Academic Scholars Award from the Department of Psychiatry, University of Toronto.

Please state what role the funders took in the study. If the funders had no role, please state: "The funders had no role in study design, data collection and analysis, decision to publish, or preparation of the manuscript."
If this statement is not correct you must amend it as needed.
Please include this amended Role of Funder statement in your cover letter; we will change the online submission form on your behalf.

RESPONSE: Statement was add in the manuscript (page 10) and the cover letter.

4. Thank you for stating the following in the Competing Interests section:
SCC has received research funding and test materials from Nestlé Health Science. He consults for Nestlé Health Science and Cerecin. OKF reports speaker fees from Lundbeck Pharma A/S, consultant work for WCG International, and serving on an advisory board for Boehringer-Ingelheim. BE is part of the Advisory Board of Boehringer Ingelheim, Lundbeck Pharma A/S; and has received lecture fees from Boehringer Ingelheim, Otsuka Pharma Scandinavia AB, and Lundbeck Pharma A/S. SMA has received honoraria from HLS therapeutics and Boehringer Ingelheim. MH has received consultant fees from Alkermes and MERCK. The other authors declare that the research will be conducted in the absence of any commercial or financial relationships that could be construed as a potential conflict of interest.

Please confirm that this does not alter your adherence to all PLOS ONE policies on sharing data and materials, by including the following statement: "This does not alter our adherence to PLOS ONE policies on sharing data and materials.” (as detailed online in our guide for authors [http://journals.plos.org/plosone/s/competing-interests](https://can01.safelinks.protection.outlook.com/?url=http%3A%2F%2Fjournals.plos.org%2Fplosone%2Fs%2Fcompeting-interests&data=05%7C02%7CMelanie.Fortier2%40USherbrooke.ca%7C7ff38b5010de4c2aed0408dd757b5763%7C3a5a8744593545f99423b32c3a5de082%7C0%7C0%7C638795894821095417%7CUnknown%7CTWFpbGZsb3d8eyJFbXB0eU1hcGkiOnRydWUsIlYiOiIwLjAuMDAwMCIsIlAiOiJXaW4zMiIsIkFOIjoiTWFpbCIsIldUIjoyfQ%3D%3D%7C0%7C%7C%7C&sdata=4BdREiu43WTmubl%2Bh6JLtd6ChXCGnOyOckREDaQPnkY%3D&reserved=0)). If there are restrictions on sharing of data and/or materials, please state these. Please note that we cannot proceed with consideration of your article until this information has been declared.

Please include your updated Competing Interests statement in your cover letter; we will change the online submission form on your behalf.

RESPONSE: Statement was add in the manuscript (page 10) and the cover letter.

5. Your ethics statement should only appear in the Methods section of your manuscript. If your ethics statement is written in any section besides the Methods, please delete it from any other section.

 RESPONSE: We have revised the manuscript to ensure the ethics statement appears only in the Methods section, as requested.

 6. Please provide a complete Data Availability Statement in the submission form, ensuring you include all necessary access information or a reason for why you are unable to make your data freely accessible. If your research concerns only data provided within your submission, please write "All data are in the manuscript and/or supporting information files" as your Data Availability Statement.

- Je ne me rappelle pas si c’était fait. Le « submission form » doit être dans le site internet ?

 7. Please include captions for your Supporting Information files at the end of your manuscript, and update any in-text citations to match accordingly. Please see our Supporting Information guidelines for more information: http://journals.plos.org/plosone/s/supporting-information.

RESPONSE: We have ensured that the guidelines for Supporting Information have been followed.

8. Please review your reference list to ensure that it is complete and correct. If you have cited papers that have been retracted, please include the rationale for doing so in the manuscript text, or remove these references and replace them with relevant current references. Any changes to the reference list should be mentioned in the rebuttal letter that accompanies your revised manuscript. If you need to cite a retracted article, indicate the article’s retracted status in the References list and also include a citation and full reference for the retraction notice.

RESPONSE: We have reviewed our reference list to ensure it is complete and correct. No retracted articles are cited.
